# Physician Attitudes Embedded Within Electronic Medical Records of Persons With and Without Serious Mental Illness

**DOI:** 10.1093/schbul/sbag004

**Published:** 2026-03-21

**Authors:** Karly A Murphy, Maria I Grajeda Martinez, Lisa Young, Brant Chee, Gail L Daumit, Mary Catherine Beach

**Affiliations:** Division of General Internal Medicine, Department of Medicine, University of California, San Francisco, San Francisco, CA 94115, United States; Department of Medicine, University of Texas Southwestern Medical School, Dallas, TX 75390, United States; Department of Medicine, Johns Hopkins University School of Medicine, Baltimore, MD 21205, United States; Applied Physics Laboratory, Johns Hopkins University, Baltimore, MD 20723, United States; Division of General Internal Medicine, Johns Hopkins School of Medicine, Baltimore, MD 21205, United States; Division of General Internal Medicine, Johns Hopkins School of Medicine, Baltimore, MD 21205, United States; Berman Institute of Bioethics, Johns Hopkins University, Baltimore, MD 21205, United States

**Keywords:** mental illness, stigma, bias, language, internal medicine, electronic health records

## Abstract

**Background and Hypothesis:**

Negative descriptors and stigmatizing language of patients have been found in electronic medical records. Using electronic health records, this study sought to describe language patterns that reflected positive or negative attitudes for patients with and without a serious mental illness.

**Study Design:**

Content analysis was performed on ambulatory internal medicine progress notes from patients with serious mental illness (schizophrenia, bipolar disorder, major depression with psychosis; *n* = 511) and a control population (*n* = 511), matched on age, sex, and race. Fisher’s exact test was used to compare frequencies of identified themes across groups.

**Study Results:**

Language reflecting negative attitudes appeared at greater frequency in notes of patients with serious mental illness than without (18.0% vs 11.7%, *P* = .006). Language expressing physicians’ frustration (5.7% vs. 2.2%, *P* = .005) and questioning patient’s credibility (5.1% vs. 1.8%, *P* = .0005) also appeared more frequently in notes for patients with serious mental illness (vs. without). Language reflecting positive attitudes appeared at similar rates (37.0% vs. 34.4%, *P* = .4). Compliments or approval of patients (16.8% vs. 8.0%, *P* < .001) or shared decision-making statements (8.2% vs. 4.7%; *P* = .03) were more frequent among patients with mental illness than without.

**Conclusions:**

Physicians may have differential emotional engagement—positive and negative—during visits with patients with serious mental illness. While not common, negative language and questioning patient credibility occurred more frequently in notes for patients with SMI than without. Given the stigma surrounding mental illness, work is needed to understand whether negative attitudes conveyed in notes are associated with poorer quality of care.

## Introduction

Populations with serious mental illness, such as schizophrenia or bipolar disorder, experience premature mortality and die 10-20 years earlier than the general population.^1^^,^^2^ They are also less likely to receive guideline concordant-care care for acute and chronic medical conditions.^3-7^ Prior work suggests that clinician-level factors, including lack of time on clinicians’ part, and stigma, may contribute to lower rates of guideline-concordant care for populations with serious mental illness.^8^^,^^9^

Implicit bias refers to the unconscious activation of stereotypes, which can shape individuals’ thought processes, decision-making, and actions.^10^ According to sociologists Link and Phelan, stigma arises from these stereotypes about a group, leading to negative attitudes and discriminatory behaviors toward that group.^11^ Within clinical practice, researchers have documented evidence of negative attitudes and stigmatizing language about patients within the electronic medical record. Such language has been shown to impact future clinician attitudes and decision-making, thereby propagating stigma in healthcare settings.^12-15^ Furthermore, implicit bias among healthcare professionals has been associated with disparities in the quality of care provided to racial and ethnic minority populations.^16-18^

Mental illness being seen as a stigmatized condition, yet previous studies have used survey data and clinical vignettes to examine healthcare providers’ stigmatizing attitudes and behaviors toward populations with mental illness.^19-21^ For example, general medical providers who held more stigmatizing attitudes about patients with mental illness were more likely to believe that patients with mental illness would not adhere to treatment and were less likely to refer to a specialist or refill a medication.^19-21^ Moreover, patients with mental illness also report being the recipient of stigma from healthcare providers.^22-24^ Despite the fact that stigmatizing language has no place in medical documentation, there is limited understanding of the use of stigmatizing attitudes in clinical documentation for patients with a serious mental illness. In this study, we sought to identify whether differential language patterns were used in electronic medical record notes for people with serious mental illness compared with those without serious mental illness.

## Methods

We conducted a mixed methods study to identify ways in which clinicians express positive and negative attitudes in patients’ medical records and then quantified and compared the prevalence of these attitudes in the records of patients with and without serious mental illness.

### Study Population

We abstracted 10 500 progress notes from an ambulatory internal medicine clinic at an urban academic medical center written by attending and internal medicine resident physicians in 2017. Data abstracted included demographic data of patients but did not include demographic data on physicians.^12^ All progress notes were from patients who were established at a primary care clinic.

From this larger collection of patient notes, we initially identified 570 unique patients with serious mental illness, defined as schizophrenia, schizoaffective disorder, bipolar disorder, or major depressive disorder with psychosis. We identified 219 patients using encounter ICD-10 billing codes for serious mental illness (f20.x, f25.x, f30.x-f31.x, f33.3) and 351 patients from note-based text. We chose to include diagnoses from note-based text, such as the opening “one-liner” sentence, as the goal of the one-liner in internal medicine is to convey a brief informative statement about the patient (eg, a 43-year-old male with a history of schizophrenia) as part of a clinical case presentation.^25^ The reader of the note may not necessarily have access to additional information to confirm the accuracy of the mental illness diagnosis, relying solely on the note for clinical details. However, the way a mental illness is described in the note could lead to the assumption that the patient has a mental illness, even if the diagnosis is incorrect.

After manual review of the entirety of the note-based texts, 59 patients were excluded as the serious mental illness diagnosis was not clearly associated with the patient of record (eg, father had a history of schizophrenia, bipolar disorder listed within differential without definitive diagnosis); thus, our sample size for patients with serious mental illness was 511. We identified a control population of 511 unique patients, matched 1:1 on age, sex, and race. Individuals in the control population were mutually exclusive from the population with a serious mental illness. All patients included in the exposure and control groups were returning for routine primary care, which could chronic disease management, preventive care and screening, or acute health concerns as would be expected in a primary care clinic. For each unique patient, we randomly selected one encounter note for review. The reason for the clinical visit was not considered when the random note was selected. All extracted notes were stored in a secure virtual environment, and only study team members had access to the records. The [blinded for review] institutional review board approved this study with a waiver of informed consent.

### Analytic Plan

Our research team included 3 physicians, 1 medical student, 1 research assistant, and 1 computer scientist. For each encounter, the entirety of the clinical note was manually reviewed. We performed content analysis of the unstructured, free-text section of patient medical records.^12^ Content analysis is a qualitative method that examines the characteristics and patterns of language as a form of communication, emphasizing the contextual meaning of the text, and categorizes the large amounts of text into themes and patterns the reflect shared meanings.^26^

An initial codebook of themes describing positive and negative language was based on prior work from the study team.^12^ Two authors (M.G., K.M.) then analyzed 100 notes, a process where they independently read and abstracted text that reflected positive and negative attitudes from the same notes (“double-coding”). They focused on free text that documented instances where the author communicated emotion or the patient or clinical scenario could potentially affect the reader’s perspective about the patient.^27^^,^^28^ Differences in coding were iteratively discussed with a third reviewer (M.C.B.) until all study team members reached consensus on coding. If investigators identified an additional theme using an inductive approach, the theme was added to the codebook. Refinements to the codebook were discussed by the team, and this iterative process was repeated twice to refine codes and themes. Themes were refined and consolidated within the existing framework of positive and negative attitudes. The codebook was then reviewed and refined by the entire team, and a finalized list of codes was created. All remaining notes were then coded by at least two authors (M.G., K.M., L.Y.). Any differences between coders were addressed by iterative group discussion (M.G., K.M., L.Y., M.C.B.) until consensus was reached. We used NVivo, version 11 for the qualitative analysis.

As part of this exploratory analysis, Fisher’s exact test was used to compare whether the frequencies of themes were different between groups with and without serious mental illness. Themes were considered present if identified at least once within a clinical encounter. If a theme was present multiple times, it was not used in comparative analyses as there was concern that variation may reflect the physician’s writing style. We used Stata, version 16 for statistical analyses.

## Results

### Study Participants

A total of 1022 clinical encounter notes from 142 physicians were reviewed. Each clinical encounter note was for a unique patient, with a total of 511 patients with serious mental illness and 511 patients without serious mental illness. The majority of patients were female (*n* = 610, 59.7%) and Black (*n* = 833, 81.5%), with a mean age of 57.2 years (SD = 16.2). As participants with and without serious mental illness were selected using a matching process, no statistical difference was observed by demographics across groups.

### Negative Language

We examined four categories of negative language: (1) physician frustration or negative emotion; (2) questioning patient credibility; (3) physician authority; (4) non-adherence without an explanation (Table 1). These categories were not mutually exclusive. We identified a total of 152 (14.9%) encounter notes with at least one excerpt representing negative language. More encounter notes for patients with serious mental illness contained negative language (*n* = 92, 18.0%) compared with encounter notes for patients without serious mental illness (*n* = 60, 11.7%) (*P* = .006).

**Table 1:** Negative Attitudes and Language Categories

**Categories**	**Definition**	**Sample excerpts**	**SMI,** ***N* (%)**	**Non-SMI,** ***N* (%)**	***P*-value**
Physician frustration or negative emotions	Disapproving of patient’s actions or negative feelings for patient	“She continues to refuse a statin or beta blocker for her history of CAD. I told her that as long as she quit smoking, I wouldn’t fight her on this, but to consider that these medications can lower overall mortality.” (SMI) “Again asked about ostomy reversal, and I again stated he would need to see Surgery in clinic to discuss if and when that is possible (referral given at last visit). He was provided with Surgery clinic’s name and phone number.” (SMI) “All this despite extensive lifestyle counseling in the past. I again reiterate diet/exercise/weight loss, but we clearly failed these interventions.” (Non-SMI) “She did not know that she needed to have orthopedic referral despite being told several times.” (Non-SMI)	29 (5.7)	11 (2.2)	.005
Questioning of the patient’s credibility	Disbelief in what patient said or points out inconsistencies in patient experience, history, symptoms.	“My guess is she consume more carbohydrates than she lets on.”(Non-SMI) “She reports that she has been taking all of her medications up until the night before last. Her son, however, reports that she has not had any medications in 4 months.” (Non-SMI) “One month ago he was given 90 tabs of oxycodone. He stated he is using one per day, with dressing changes, but also stated he had only 4 tabs remaining. When I pointed out this discrepancy, he stated that perhaps he takes oxycodone twice per day. Even still, he should have 30 tabs remaining.” (SMI) “Acutely, I am concerned that she is telling me she occasionally takes entire bottles of tylenol at once. It’s unclear as to whether she’s being truthful (her therapist didn’t think she was), but I was very emphatic that she should not do this and could die in the future.” (SMI)	26 (5.1)	9 (1.8)	.005
Emphasizing physician authority	Physician using language to emphasize authority over patient.	“I have told her that the #1 thing she can do for herself to prolong her lifespan is to quit [smoking].” (SMI) “She was provided strict instructions to take her medications as her Ab1c increased by 5pts in the setting of noncompliance.” (Non-SMI) “Ms. X and her daughter in law was provided with strict instructions the she should be on either fluticasone (Flovent) OR fluticasone-salmeterol (Advair)- but NOT both.” (Non-SMI)	4 (0.8)	2 (0.4)	.7
Non-adherence to medication or treatment plan	Reporting of patient not following medication regimen or plan	“She reports she has stopped taking pravastatin for undetermined reasons.” (SMI) “I’m concerned for undertreatment since she did not take the medication on five consecutive days.” (SMI) “With respect to his HLD, the patient is not currently taking any medications. He has been on atorvastatin in the past, which the patient discontinued on his own.” (Non-SMI) “She has not been taking her lantus and so I recommended restarting.” (Non-SMI)	50 (9.8)	46 (9.0)	.7

#### Physician Frustration or Negative Emotions

Language reflecting frustration and irritation from the perspective of the physician and directed towards the patient and their actions or lack of action appeared in primary care notes. Often, physicians emphasized the misalignment between the patient and the medical provider by their word choice (“Today’s visit was limited by Mr. [name]’s anger and pre-occupation with his surgical history”). Excerpts suggested a continuation of a previous discussion (eg, “again,” “really,” or “reiterated”) or placed blame on the patient as being unreasonable. For example, note excepts included, “I am again recommending metformin at this time [as] it is [the] recommendation (with clomiphene) for women who are actively trying to conceive…..however she is still against taking this” or “We again focused today’s visit on SMOKING CESSATION.” In the latter excerpt, the use of the word “again” coupled with the sudden use of capital letters suggested negative emotional intensity that this clinical topic was repeated. When the outcome differed from what the physician deemed appropriate or that the physician had tried to do everything that they thought was appropriate, blame appeared to be transferred to the patient (“Really needs to be on insulin but has consistently refused this and does again today” or “despite multiple calls, she never obtained these labs”). These statements were observed more frequently in notes for patients with serious mental illness (5.7%) versus without mental illness (2.2%) (*P* = .005).

#### Questioning Patient Credibility

Physicians conveyed a degree of disbelief or noted inconsistencies within the patient’s history. For instance, in one note, the physician wrote, “She reports she has been taking all her medications up until the night before last. Her son, however, reports that [she] has not had any medications in 4 months.” In another note, a physician states, “Mr. X has poorly controlled hypertension. This has been a longstanding problem even in the past when he reported compliance with all his medications (though I have questioned for some time whether he has ever been compliant or not).” Physicians expressed disbelief more for patients with serious mental illness versus without serious mental illness (5.1% vs. 1.8%; *P* = .0005).

#### Emphasizing Physician Authority

In a few encounters, physicians used language to convey their authority as the definitive approach. Phrases that physicians wrote, such as “I instructed her to” or “I have told her again” provided a sense that the patient was viewed as less autonomous in their physician-patient relationship and required more strict instructions to enact a plan. Authoritative language was used in similar frequencies across groups (0.8% vs 0.4%; *P* = .7).

#### Non-adherence to Medication or Treatment Plan without Explanation

Physicians also noted when a patient did not follow a treatment plan and did not include a context or explanation for these events. For example, a physician wrote “poor compliance with meds,” or “is not taking coreg or hydralazine and instead went back to terazosin,” or “Dr. S ordered Utox monthly for one year on 9/5; none of these have been done.” Most statements focused on medication adherence, but others referenced the need for imaging or follow-up testing as part of an existing diagnostic evaluation. Frequency of these sentiments did not differ across groups (9.8% vs 9.0%; *P* = .7).

### Positive Language

We identified four categories of positive language: (1) physician approval or positive emotions; (2) collaborative decision-making; (3) minimization of blame; (4) physician concern for their patient (Table 2). These categories were not mutually exclusive; over one-third of encounter notes (*n* = 365, 35.7%) contained at least one excerpt representing positive language. Notes with at least one positive language excerpt occurred at similar frequencies for patients with and without serious mental illness (37.0% vs. 34.4%; *P* = .4).

**Table 2:** Positive Attitudes and Language Categories

**Categories**	**Definition**	**Sample excerpts**	**SMI,** **%**	**Non-SMI,** **%**	***P*-value**
Physician approval or positive emotions	Approving of patient actions or positive emotions towards patient/situation	“He has made incredible progress as evidenced by his change in his HbA1C.” (SMI) “I am happy to say she is doing well with her current regimen for her anxiety and insomnia.” (SMI) “He also notes that he is interested in quitting smoking, and I am encouraged him to this effect.” (Non-SMI) “It is very encouraging that she is interested in pursuing risk factor modification.” (Non-SMI)	86 (16.8)	41 (8.0)	<.001
Collaborative decision-making	Both physician and patient understand and agree of health plan and	“Discussed potentially starting nicotine lozenges, which he was amiable to trying.” (SMI) “We have discussed the role of statins in risk reduction, and for the meantime are going to focus on smoking cessation as primary prevention.” (SMI) “We agreed to work on weight loss goals together and better medication adherence.” (Non-SMI) “Our one shared goal is for her to improve her diet and lose weight.” (Non-SMI)	42 (8.2)	24 (4.7)	.03
Minimization of Blame	Acknowledgment of situations outside the patients control that have an impact in patient’s health or health plan	“He did not take the gabapentin because he was worried about sedation.” (SMI) HE has not picked up his medication due to cost and states he was charged $96 for his medications.” (SMI) “She did not undergo a scheduled screening colonoscopy due to inability to take prep.” (Non-SMI) “He reports that he ran out of his Lasix and warfarin on Monday and has not been able to fill it due to cost.” (Non-SMI)	79 (15.1)	98 (19.2)	.097
Physician Concern for Patient	Physician expresses empathy for patient/situation that affects patient health	“I would like her to get this cataract taken care of as I feel as though it is significantly affecting her vision and her balance putting her at higher risk for a fall. I discussed with her that the last thing I want is for her to have a setback like a hip fracture.” (SMI) “I provide my recommendation to his girlfriend should he wish to seek further care.” (SMI) “In regards to his frequent falls at home, I am concerned because they have happened twice in the past few months.” (Non-SMI) “We called down to pharmacy today to switch his Advair (tier 2) to Pulmicort (tier 1) which will make this much more affordable to him.” (Non-SMI)	53 (10.4)	50 (9.8)	.8

#### Physician Approval or Positive Emotions

Physicians directly complimented their patients or provided explicit approval of their patients’ actions and self-management of chronic disease. For example, physicians noted, “congratulated her on excellent dietary choices” or “her daily walking has helped her lose some intentional weight, which has helped her [blood pressure] as well.” Some notes reflected the physician’s optimism or positive emotions around the clinical encounter itself (“our appointment today is markedly better than our previous visits”). These approving statements were noted more frequently for patients with serious mental illness than without (16.8% vs. 8.0%; *P* < .001).

#### Collaborative Decision-Making

Physicians described how the patient and the physician mutually agreed on a treatment plan. For example, a physician wrote, “Mr. X and I discussed how he can attain better control of diabetes, including more frequent [blood glucose] checks at home. I asked him to identify any obstacles to this and he believes there are none and he can manage this.” This category included encounters where the physician and patient initially had differing opinions and came to a consensus. For example, a physician wrote, “He still has constant back pain, which unfortunately is not well controlled with oxycodone 20mg bid. We discussed why I do not suggest increasing oxycodone dose as has a hx of substance abuse and has constipation/gastroparesis.” Physicians included statements about collaborative decision-making more often for patients with serious mental illness than without serious mental illness (8.2% vs. 4.7%; *P* = .03).

#### Minimization of Blame

We also identified encounters where physicians included a reason why the patient was unable to adhere to a proposed treatment plan. This included medication nonadherence where an explanation was given for the nonadherence. Physicians specifically identified if a new problem occurred that impacted the patient’s ability to complete the task (“For example, one physician wrote “He stopped taking Lyrica due to mood changes”). Others noted environmental contexts that impacted care delivery (“She states she missed appointments because her reminder notices were sent to her apartment, where mail is often stolen, and no calls come through to her phone”). These phrases suggested physician empathy, in contrast to statements where no explanatory reason was given for non-adherence. Physicians provided these explanatory statements to describe why a treatment plan was not adhered to at similar frequencies between groups (15.1% vs. 19.2%; *P* = .097).

Within this category, physicians also explicitly mentioned social determinants of health as the reasons why patients had nonadherence. Costs (“Shingles vaccine note yet obtained due to cost”) and insurance (“She is interested in Chantix but there are insurance issues”) were cited the most frequently. Physicians noted these social factors at similar frequencies between groups (11.2% vs. 11.9%; *P* = .8).

#### Physician Concern for their Patient

Physicians expressed statements of empathy and concern for their patients’ wellbeing, diagnoses, and challenging life situations. Sometimes, they directly acknowledged the barriers that patients faced (“I think we need to have a hard look at the patient (and her caregiver’s) priorities to reach something that is sustainable and realistic”) or other medical/psychiatric-related barriers (“The issue I’m most concerned about is her psychiatric illness. I worry that her bipolar is untreated. She has pressure speech, labile affect, tangibility”). Other examples described how the physician sought to find solutions for a problem (“He also cannot tolerate large pills. I suggested a combination pill… and confirmed with his pharmacy that the pill is not any larger than his current lisinopril dose”). These statements were observed at similar frequencies across groups (10.4% vs. 9.5%; *P* = .8).

## Discussion

Our study observed that primary care physicians conveyed negative sentiments more often in notes of patients with serious mental illness than without, specifically in the context of frustration or questioning the patients’ credibility. While using positive sentiments at similar frequencies, physicians were more likely to express approval or highlight a shared decision-making approach in medical records of patients with serious mental illness. Our results are a preliminary look and suggest that physicians may have more emotional engagement, both positive and negative, with patients who have serious mental illness. These sentiments and attitudes suggest that clinicians reading the medical record will obtain both a clinical and emotional picture of the patient.

While we cannot determine why negative descriptive language may be found in the medical record, our study is a preliminary look at possible differences in the use of negative descriptors in electronic medical records among populations with and without a serious mental illness. While our reported rates of use of negative language in documentation were low, clinicians may avoid using overtly negative language as part of professionalism and upholding ethical standards. Standardized note formats may further encourage the focus on clinical, objective details (eg, physical exam, emphasis on prior testing). Additionally, stigmatizing attitudes may be less likely to appear in formal written communication, highlighting the need to study both written and verbal interactions. In addition, negative content may not always reflect implicit biases and stigma towards populations with mental illness, it does parallel the disproportionate use negative language that is seen in other vulnerable and at-risk populations that experience implicit biases, including non-Hispanic Black patients, patients with sickle cell anemia, and patients with substance use disorders.^13^^,^^14^^,^^29^^,^^30^ For example, studies demonstrated over a two-fold increase in negative descriptive language in notes regarding Black patients compared with notes regarding white patients.^13^^,^^14^ Moreover, notes for patients with greater severity of diabetes were also found to have more stigmatizing language.^13^

Importantly, the observed negative language focused on patient behavior, such as medication adherence or lack of action. By describing interactions where the physician reiterated a message (“told again” or “despite the fact”), the concern is that the note writers are implying that the patient was exacerbating, ignorant, adversarial, or uninterested in their health. Similarly, language undermining a patient’s credibility raises concerns that the physician note writer did not trust the patient.^31^ Given that patients with serious mental illness report feeling stigmatized in healthcare settings, future work should explore whether use of negative language excerpts correlate with the presence of implicit biases from physicians.^32^^,^^33^

Diagnostic evaluations require a degree of skepticism and gathering of collateral information, and clinicians need to ask and document adherence patterns. Medical societies recommend asking about adherence and discussing barriers to optimize long-term care and outcomes.^34^ Importantly in our analysis, we sought to understand whether the clinician had obtained information that may influence changes in treatment care plans. Therefore, we classified excerpts where the note writer described nonadherence without a stated reason (“nonadherence” negative language) separately from nonadherence with a stated reason (“minimization of blame” positive language). However, it is possible that clinicians were less interested or had additional challenges when delving into why patients had nonadherence behaviors, which were coded as negative content in our analysis. Primary care physicians have reported greater challenges with taking a complete history from patients with serious mental illness who provide more tangential answers and that visits take longer.^35-37^ Given that patients with serious mental illness face greater social and structural barriers to care, including lower health literacy, low educational attainment, lower socioeconomic status^32^ future work is needed to support physicians to ask patients with mental illness about their barriers to care and then to address these barriers.

Similarly, the greater frequency of comments expressing approval within notes of patients with serious mental is intriguing. Phrases of “I am happy to say” or “congratulated her on excellent dietary choices” interjects the physician’s opinion and judgement into the narrative. With one perspective, it speaks to a physician being proud of a patient’s progress towards their health goals or feeling optimistic for the patient. Yet in another, more cynical perspective, it could hint at a patronizing, paternalistic attitude of the physician. Paternalism occurs when the clinician holds beliefs that the patient lacks the capacity or ability to make the “right” decision.^38^ Given the historical and societal trends of patients with mental illness feeling stigmatized and marginalized by the healthcare system, future work should continue to examine these more positive attitude phrasing and explore their use and context. It is our hope that all clinicians respect patient autonomy and hold patients in true high regard.

Our work provides a nuanced and complex view of the interactions between physicians and patients with serious mental illness. Through language patterns, we recognized the emotional energy and engagement that physicians had during clinical interactions with patients. Just as we found positive examples of emotional engagement, such as approval and concern for patients, we also found negative emotions present, such as frustration or incredulity. The presence of emotional engagement is not itself problematic, but rather it is important to highlight whether a negatively charged emotional exchange could influence current and future clinicians. With the rise of artificial intelligence-based scribes for medical documentation,^39^ care is needed to ensure that large language models also do not perpetuate negative attitudes. As natural language processing methods are increasingly used to study electronic health records,^13^^,^^14^^,^^29^^,^^40^ manual chart review may best reflect how notes are used in clinical practice.

Negative sentiments may represent challenges stemming from a clinical encounter and not implicit biases or stigma. The examples highlighted by physicians expressing frustration may simply be physicians frustrated with having a conversation again, such as around chronic disease management. Similarly, use of capital letters (eg, “SMOKING CESSATION”) may be simply emphasis on a clinical topic and not meant to represent shouting or condescension. Yet the concern is that negative language depicts the patient as unreasonable or uninterested in their own healthcare may reflect physicians’ implicit biases being confirmed. Moreover, in the age of open access to notes, patients have electronic access to their clinicians’ visit notes. Not only can patients see potential negative sentiments (or worse, stigmatizing language), but also that these clinical notes are the legal medical record. These statements could further perpetuate bias and disenfranchise patients with mental illness.

Future work may wish to focus on combating stigma and supporting clinicians in caring for populations with serious mental illness.^41^ Moreover, work is needed to explore potential comparisons between individuals with serious mental illness and those with less severe or subclinical mental health conditions to further elucidate the spectrum of stigmatizing attitudes. Patients with serious mental illness are more likely to experience disorganized thought processes, cognitive dysfunction, and low health literacy, which may impact disease self-management.^32^^,^^42^^,^^43^ Medical providers may need additional training in motivational interviewing, an evidence-based strategy for health behavior change and medication management for populations with and without serious mental illness.^44^ In addition, patients with serious mental illness are more likely to experience poverty, disability, and other markers of social vulnerability that negatively impact health status and access to care.^42^^,^^45^^,^^46^ These notes were drawn from an urban clinic that serves a marginalized population, and it is important that physicians have accessible resources to address these social needs.

### Limitations

Our study has several limitations. First, data were collected from one urban ambulatory internal medicine clinic at an academic medical center, which may limit its generalizability. Use of an inductive qualitative analysis strategy and the initial codebook categories were drawn from prior work,^12^ but may have made it more challenging to identify differences between groups given the subjective nature of qualitative analysis. Yet, notes from study participants were from a majority Black patient population, and Black race has been associated with greater frequency of negative attitudes by clinician note writers into the electronic health record.^13^^,^^14^^,^^29^ While we matched participants, residual confounding may remain. Second, we do not have individual-level data for the physician note writers, including demographics and level of training (attending or medicine resident). Level of clinical experience has been associated with less stigmatizing language^13^ and may be a source of future work. Third, notes were written in 2017 when patients did not have open access to clinical records. We do not know the extent to which physicians may change their documentation habits based on this. Future work is needed to examine whether open access to clinical records shift use of negative language, particularly toward those with serious mental illness and other marginalized populations. However, to our knowledge, this is the first preliminary study examining difference in language patterns regarding patients with and without a serious mental illness. Fourth, notes were of variable lengths; longer notes inherently contain more content, which may increase the likelihood of identifying negative themes. We did not include a measure of note length in our analysis. However, notes were often coded without knowing whether the note referenced a patient with serious mental illness, and note length may reflect external factors, such as time available to the patient or physician or the clinical content that needed to be addressed during the encounter. Fifth, we identified individuals with a serious mental illness using both ICD codes and text-based descriptors, which may have misclassified individuals in the exposure group. However, our interest is whether the clinician note writer perceived the patient to have a mental illness and whether there may be differences in language patterns. Moreover, prior work suggests low concordance of diagnostic information of a serious mental illness between primary care and inpatient hospitalizations.^47^ The siloing of care between psychiatry and primary care has created barriers in verifying diagnoses.^48^ Finally, our research team was unable to verify the true attitudes and assumptions of the clinicians who wrote the notes. Our findings may include unverifiable assumptions of the note writers. We were also unable to account for differences in the writing styles of clinicians who may have included complimentary language differently.

## Conclusions

Populations with mental illness are historically marginalized within society, with high health care needs and premature mortality from physical health conditions. Serious mental illness, such as schizophrenia and bipolar disorders, are stigmatized conditions that may influence how healthcare providers view and engage with patients. We found that notes of patients with serious mental illness were more likely to contain language expressing frustration and incredulity, but also complimentary and shared-decision language compared with notes from patients without serious mental illness. These differences suggest that primary care physicians are emotionally engaged, both positively and negatively, with patients with serious mental illness. Positive engagement can foster trust and improve treatment adherence, enhancing the quality of care.

Given the stigma surrounding serious mental illness, it is crucial to investigate whether negative attitudes conveyed in notes are associated with poorer quality of care. Additionally, this work suggests greater awareness and training for clinicians are needed when communicating about and caring for patients with serious mental illness. Implementing best practices, such as reflective writing and peer reviews of clinical notes, could help mitigate bias and prevent the medical record from adversely influencing future clinicians who will care for patients with serious mental illness.

## References

[1] Olfson M, Gerhard T, Huang C, Crystal S, Stroup TS. Premature mortality among adults with schizophrenia in the United States. *JAMA psychiatry*. 2015;72:1172-1181. 10.1001/jamapsychiatry.2015.1737 26509694

[2] Colton CW, Manderscheid RW. Congruencies in increased mortality rates, years of potential life lost, and causes of death among public mental health clients in eight states. *Prev Chronic Dis*. 2006;3:A42. 16539783 PMC1563985

[3] McGinty EE, Baller J, Azrin ST, Juliano-Bult D, Daumit GL. Quality of medical care for persons with serious mental illness: a comprehensive review. *Schizophr Res*. 2015;165:227-235. 10.1016/j.schres.2015.04.010 25936686 PMC4670551

[4] Domino ME, Beadles CA, Lichstein JC, et al. Heterogeneity in the quality of care for patients with multiple chronic conditions by psychiatric comorbidity. *Med Care*. 2014;52:S101-S109. 10.1097/mlr.0000000000000024 24561748

[5] Domino ME, Wells R, Morrissey JP. Serving persons with severe mental illness in primary care–based medical homes. *Psychiatr Serv*. 2015;66:477-483. 10.1176/appi.ps.201300546 25686809

[6] Olesiuk WJ, Farley JF, Domino ME, Ellis AR, Morrissey JP. Do medical homes offer improved diabetes care for Medicaid enrollees with co-occurring schizophrenia? *J Health Care Poor Underserved*. 2017;28:1030-1041. 10.1353/hpu.2017.0094 28804075 PMC5826756

[7] Swietek KE, Domino ME, Beadles C, et al. Do medical homes improve quality of care for persons with multiple chronic conditions? *Health Serv Res*. 2018;53:4667-4681. 10.1111/1475-6773.13024 30088272 PMC6232445

[8] Fortuna KL, Williams A, Mois G, Jason K, Bianco CL. Social processes associated with health and health behaviors linked to early mortality in people with a diagnosis of a serious mental illness. *Perspect Psychol Sci*. 2021;17:183-190. 10.1177/1745691621990613 34264159 PMC8760359

[9] Thornicroft G, Mehta N, Clement S, et al. Evidence for effective interventions to reduce mental-health-related stigma and discrimination. *Lancet.* 2016;387:1123-1132. 10.1016/s0140-6736(15)00298-6 26410341

[10] Greenwald AG, Banaji MR, Rudman LA, Farnham SD, Nosek BA, Mellott DS. A unified theory of implicit attitudes, stereotypes, self-esteem, and self-concept. *Psychol Rev*. 2002;109:3-25. 10.1037/0033-295x.109.1.3 11863040

[11] Link BG, Phelan JC. Conceptualizing stigma. *Annu Rev Sociol*. 2001;27:363-385. 10.1146/annurev.soc.27.1.363

[12] Park J, Saha S, Chee B, Taylor J, Beach MC. Physician use of stigmatizing language in patient medical records. *JAMA Netw Open*. 2021;4:e2117052-e2117052. 10.1001/jamanetworkopen.2021.17052 34259849 PMC8281008

[13] Himmelstein G, Bates D, Zhou L. Examination of stigmatizing language in the electronic health record. *JAMA Netw Open*. 2022;5:e2144967. 10.1001/jamanetworkopen.2021.44967 35084481 PMC8796019

[14] Sun M, Oliwa T, Peek ME, Tung EL. Negative patient descriptors: documenting racial bias In the electronic health record. *Health affairs (Project Hope)*. 2022;41:203-211. 10.1377/hlthaff.2021.01423 35044842 PMC8973827

[15] Goddu AP, O'Conor KJ, Lanzkron S, et al. Do words matter? Stigmatizing language and the transmission of bias in the medical record. *J Gen Intern Med*. 2018;33:685-691. 10.1007/s11606-017-4289-2 29374357 PMC5910343

[16] Nelson A . Unequal treatment: confronting racial and ethnic disparities in health care. *J Natl Med Assoc*. 2002;94:666-668. 12152921 PMC2594273

[17] Sabin JA, Greenwald AG. The influence of implicit bias on treatment recommendations for 4 common pediatric conditions: pain, urinary tract infection, attention deficit hyperactivity disorder, and asthma. *Am J Public Health*. 2012;102:988-995. 10.2105/ajph.2011.300621 22420817 PMC3483921

[18] Dehon E, Weiss N, Jones J, Faulconer W, Hinton E, Sterling S. A systematic review of the impact of physician implicit racial bias on clinical decision making. *Acad Emerg Med Off J Soc Acad Emerg Med*. 2017;24:895-904. 10.1111/acem.13214 28472533

[19] Corrigan PW, Mittal D, Reaves CM, et al. Mental health stigma and primary health care decisions. *Psychiatry Res*. 2014;218:35-38. 10.1016/j.psychres.2014.04.028 24774076 PMC4363991

[20] Stone EM, Chen LN, Daumit GL, Linden S, McGinty EE. General medical clinicians’ attitudes toward people with serious mental illness: a scoping review. *J Behav Health Services Res*. 2019;46:656-679. 10.1007/s11414-019-09652-w PMC7251232 30887413

[21] Sullivan G, Mittal D, Reaves CM, et al. Influence of schizophrenia diagnosis on providers' practice decisions. *J Clin Psychiatry*. 2015;76:1068-1074. 10.4088/JCP.14m09465 26335084

[22] Corrigan PW, Pickett S, Batia K, Michaels PJ. Peer navigators and integrated care to address ethnic health disparities of people with serious mental illness. *Social Work Public Health*. 2014;29:581-593. 10.1080/19371918.2014.893854 PMC5371355 25144699

[23] Kohn L, Christiaens W, Detraux J, et al. Barriers to somatic health care for persons with severe mental illness in Belgium: a qualitative study of patients’ and healthcare professionals’ perspectives. *Frontiers Psychiatry*. 2021;12:798530. 10.3389/fpsyt.2021.798530 PMC8825501 35153863

[24] Thornicroft G, Brohan E, Rose D, Sartorius N, Leese M, INDIGO Study Group. Global pattern of experienced and anticipated discrimination against people with schizophrenia: a cross-sectional survey. *Lancet.* 2009;373:408-415. 10.1016/s0140-6736(08)61817-6 19162314

[25] Brett AS, Goodman CW. First impressions - should we include race or ethnicity at the beginning of clinical case presentations? *N Engl J Med*. 2021;385:2497-2499. 10.1056/NEJMp2112312 34951753

[26] Hsieh HF, Shannon SE. Three approaches to qualitative content analysis. *Qual Health Res*. 2005;15:1277-1288. 10.1177/1049732305276687 16204405

[27] Raskind IG, Shelton RC, Comeau DL, Cooper HLF, Griffith DM, Kegler MC. A review of qualitative data analysis practices in health education and health behavior research. *Health Educ Behav*. 2019;46:32-39. 10.1177/1090198118795019 30227078 PMC6386595

[28] Nabi RL, Green MC. The role of a Narrative's emotional flow in promoting persuasive outcomes. *Media Psychol*. 2015;18:137-162. 10.1080/15213269.2014.912585

[29] Beach MC, Saha S, Park J, et al. Testimonial injustice: linguistic bias in the medical records of black patients and women. *J Gen Intern Med*. 2021;36:1708-1714. 10.1007/s11606-021-06682-z 33754318 PMC8175470

[30] Hirshman R, Hamilton S, Walker M, Ellis AR, Ivey N, Clifton D. Stigmatizing and affirming provider language in medical records on hospitalized patients with opioid use disorder. *J Hosp Med*. 2025;20:26-32. 10.1002/jhm.13472 39080856

[31] Beach MC, Saha S. Quoting patients in clinical notes: first, do No harm. *Ann Intern Med*. 2021;174:1454-1455. 10.7326/m21-2449 34399061

[32] Kaufman EA, McDonell MG, Cristofalo MA, Ries RK. Exploring barriers to primary care for patients with severe mental illness: frontline patient and provider accounts. *Issues Ment Health Nurs*. 2012;33:172-180. 10.3109/01612840.2011.638415 22364429

[33] Ross LE, Vigod S, Wishart J, et al. Barriers and facilitators to primary care for people with mental health and/or substance use issues: a qualitative study. *BMC Fam Pract*. 2015;16:135. 10.1186/s12875-015-0353-3 26463083 PMC4604001

[34] Choudhry NK, Kronish IM, Vongpatanasin W, et al. Medication adherence and blood pressure control: a scientific statement from the American Heart Association. *Hypertension*. 2022;79:e1-e14. 10.1161/hyp.0000000000000203 34615363 PMC11485247

[35] Daumit GL, Pratt LA, Crum RM, Powe NR, Ford DE. Characteristics of primary care visits for individuals with severe mental illness in a national sample. *Gen Hosp Psychiatry*. 2002;24:391-395. 12490340 10.1016/s0163-8343(02)00213-x

[36] Yarborough BJH, Stumbo SP, Perrin NA, Hanson GC, Muench J, Green CA. Effects of primary care clinician beliefs and perceived organizational facilitators on the delivery of preventive care to individuals with mental illnesses. *BMC Fam Pract*. 2018;19:16. 10.1186/s12875-017-0693-2 29329520 PMC5767018

[37] Murphy KA, Stone EM, Presskreischer R, McGinty EE, Daumit GL, Pollack CE. Cancer screening among adults with and without serious mental illness: a mixed methods study. *Med Care*. 2021;59:327-333. 10.1097/mlr.0000000000001499 33704103 PMC7952680

[38] Specker SL . Medical maternalism: beyond paternalism and antipaternalism. *J Med Ethics*. 2016;42:439-444. 10.1136/medethics-2015-103095 26893148

[39] Liu TL, Hetherington TC, Stephens C, et al. AI-powered clinical documentation and clinicians’ electronic health record experience: a nonrandomized clinical trial. *JAMA Netw Open*. 2024;7:e2432460. 10.1001/jamanetworkopen.2024.32460 39240568 PMC11380097

[40] Bilotta I, Tonidandel S, Liaw WR, et al. Examining linguistic differences in electronic health records for diverse patients with diabetes: natural language processing analysis. *JMIR Med Inform*. 2024;12:e50428. 10.2196/50428 38787295 PMC11137426

[41] Carr VJ, Lewin TJ, Barnard RE, et al. Attitudes and roles of general practitioners in the treatment of schizophrenia compared with community mental health staff and patients. *Soc Psychiatry Psychiatr Epidemiol*. 2004;39:78-84. 10.1007/s00127-004-0703-2 15022051

[42] Mueser KT, McGurk SR. *Schizophrenia Lancet*. 2004;363:2063-2072. 10.1016/s0140-6736(04)16458-1 15207959

[43] Lester H, Tritter JQ, Sorohan H. Patients’ and health professionals’ views on primary care for people with serious mental illness: focus group study. *BMJ*. 2005;330:1122. 10.1136/bmj.38440.418426.8F 15843427 PMC557894

[44] Swanson AJ, Pantalon MV, Cohen KR. Motivational interviewing and treatment adherence among psychiatric and dually diagnosed patients. *J Nerv Ment Dis*. 1999;187:630-635. 10.1097/00005053-199910000-00007 10535657

[45] Draine J, Salzer MS, Culhane DP, Hadley TR. Role of social disadvantage in crime, joblessness, and homelessness among persons with serious mental illness. *Psychiatr Serv*. 2002;53:565-573. 10.1176/appi.ps.53.5.565 11986504

[46] Drake RE, Frey W, Bond GR, et al. Assisting social security disability insurance beneficiaries with schizophrenia, bipolar disorder, or major depression in returning to work. *Am J Psychiatry*. 2013;170:1433-1441. 10.1176/appi.ajp.2013.13020214 23929355

[47] O'Neill B, Kalia S, Aliarzadeh B, et al. Agreement between primary care and hospital diagnosis of schizophrenia and bipolar disorder: a cross-sectional, observational study using record linkage. *PLoS One*. 2019;14:e0210214. 10.1371/journal.pone.0210214 30615653 PMC6322753

[48] Horvitz-Lennon M, Kilbourne AM, Pincus HA. From silos to bridges: meeting the general health care needs of adults with severe mental illnesses. *Health Affairs (Project Hope)*. 2006;25:659-669. 10.1377/hlthaff.25.3.659 16684729

